# Rupture of the Distal Biceps Tendon Combined with a Supinator Muscle Tear in a 51-Year-Old Woman: A Case Report

**DOI:** 10.1155/2011/515912

**Published:** 2011-08-22

**Authors:** Samir Nayyar, Martin Quirno, Saqib Hasan, Leon Rybak, Robert J. Meislin

**Affiliations:** ^1^Department of Orthopaedic Surgery, Hospital for Joint Diseases, 301 E 17th St Suite 1500, New York, NY 10003, USA; ^2^Department of Radiology, Hospital for Joint Diseases, New York, NY 10003, USA

## Abstract

Distal biceps tendon rupture is a relatively uncommon occurrence in the general female population, and to our knowledge, has not been reported in association with a supinator muscle tear. We report a case of 51-year-old woman who experienced sharp pain in her forearm and elbow after lifting a heavy object. History and physical examination raised suspicion for a distal biceps tendon rupture. MRI imaging determined a combined distal biceps tendon tear with a supinator muscle tear with subsequent confirmation at surgery. Surgical repair was performed for the distal biceps tendon only through a single incision approach using the Endobutton technique.

## 1. Introduction

Distal biceps tendon ruptures are relatively uncommon injuries that until recently were reported to occur in 3% of all biceps tendon ruptures [1]; however new data suggests that it may be closer to 10% [2]. They typically occur in males during their fourth to sixth decades [3]. The injury results from an extended arm being overstretched by an outside force (unintentional eccentric load to a flexed, shortened and contracted muscle) [4–6].

Compared to males, distal biceps tendon ruptures in females are extremely rare [7]. Due to its rare nature and often vague clinical presentation, making the correct diagnosis in females requires a high clinical suspicion and acuity. In contrast to the male's acute nature of injury, females usually present after months of persistent pain, variable discomfort to resisted supination, flexion, and palpation to distal biceps [2, 8]. 

Like the general population, the incidence of biceps tendon ruptures in females seems to be increasing over the years [2, 8, 9]. An increasing incidence in women has been attributed in part to a trend towards a more active lifestyle and participation in sports [8].

Supinator tears are extremely rare, usually occurring when the elbow is extended and forearm is unintentionally pronated while the supinator is contracted. 

Though intimately related anatomically and both engaged in supination, we were unable to find a reported association between rupture of the distal biceps tendon and a concomitant supinator muscle tear.

We present a case of a distal biceps tendon rupture and supinator muscle tear in a 51-year-old female.

## 2. Case

A 51-year-old female, right hand dominant, picked up a bag that weighed 22.7 kg with her right hand, when she suddenly heard a “pop” and experienced an immediate onset of pain in her forearm that radiated to her shoulder. She subsequently complained of worsening pain with rotational motion at her elbow and a mild deformity and ecchymosis in her upper arm. Her work and activities of daily living were restricted due to pain.

The patient's occupation is a secretary where she carries heavy boxes (11.3–13.6 kg) on a daily basis. She is athletic and has an active lifestyle, which includes going to the gym regularly. Her BMI was calculated as 23.8. She does not smoke or drink alcohol.

The patient sought care from her primary care physician initiated treatment with physical therapy. After minimal improvement, she was referred for orthopaedic care at the author's institution 3 months following index injury.

On physical examination, no swelling or ecchymosis was noted. There was a visible deformity in the antecubital fossa. Elbow range of motion was as follows: extension/flexion 0/135 (normal 0–140); active pronation/supination 90/90. She had mild weakness, 4+/5, in both flexion and supination when compared to opposite side. She also presented weakness, 4−/5, to resisted supination with positive tenderness at lateral epicondyle, medial epicondyle, and in the antecubital fossa.

Patient also presented with inability to supinate the forearm with the elbow extended, which raised suspicion for a possible tear in the supinator muscle. She had a positive Speed, positive biceps squeeze, and positive Heuter test. She was neurovascular intact upon examination.

**Figure 1:**
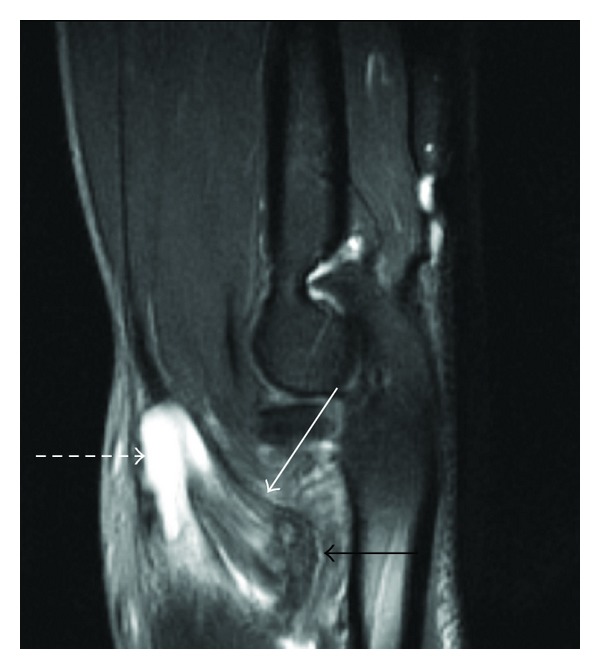
Sagittal STIR image (TR: 5900; TE: 28) of the elbow demonstrating thickening and increased solid in the distal biceps tendon consistent with high-grade partial tearing (solid white arrow). Note associated fluid in the bicipitoradial bursa (dashed white arrow) and osseous changes at radial tuberosity (solid black arrow).

**Figure 2:**
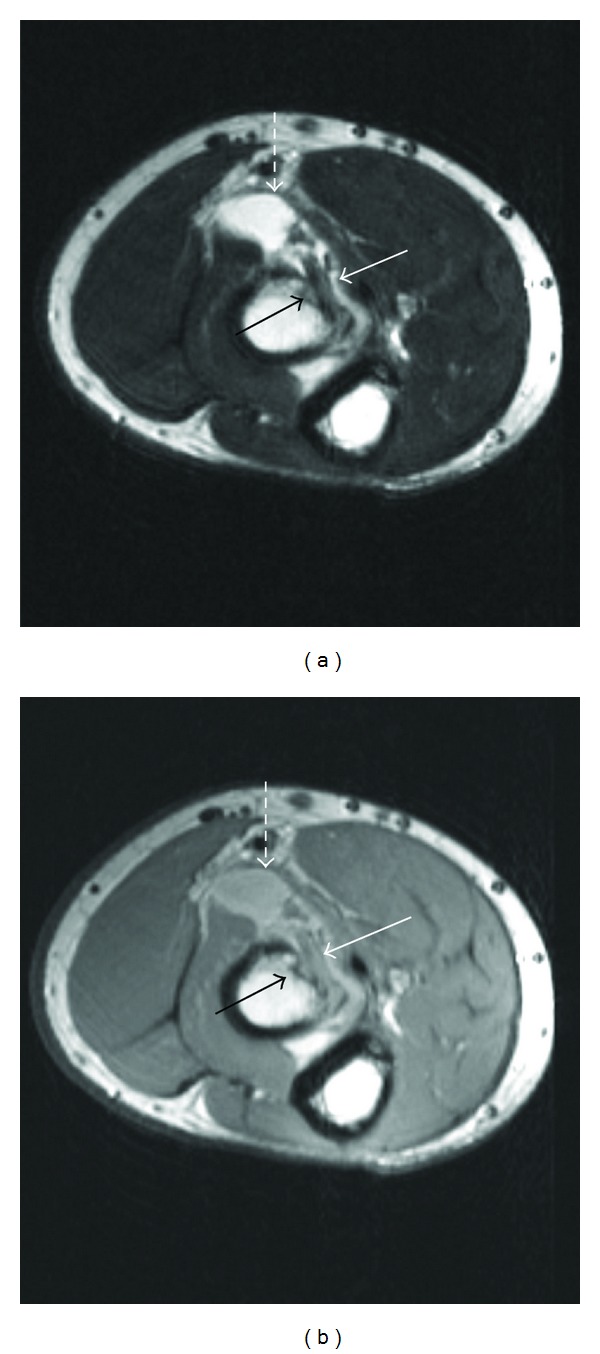
Axial T2 (TR: 3550; TE: 82) (a) and intermediate/proton density weighted (TR: 3550; TE: 16) (b) axial images at the level of the tuberosity demonstrating the high-grade partial tear of the distal biceps tendon (solid white arrow), bursitis (dashed white arrow), and osseous changes (solid black arrow).

An MRI of the elbow utilizing a closed 1.5 Tesla system (Siemens Symphony) was obtained to confirm the diagnosis. The images demonstrated marked attenuation and irregularity of the distal biceps tendon with surrounding edema and hemorrhage felt to be most consistent with a high grade partial tear. No full thickness retracted component was identified, and the lacertus fibrosus was intact (Figure 1). Also noted was focal discontinuity of the anterior fibers of the supinator muscle where the muscle appeared to be partially stripped from the underlying surface of the radial neck and displaced radially (Figures 2(a) and 2(b)). 

Plain radiographic findings were normal with no bony arthritis seen at the ulnohumeral and radiocapitellar joints; no bony avulsion fractures were seen.

## 3. Surgical Procedure

The patient had a single incision over the antecubital crease that provided direct visualization. A large pseudocyst was present at the level of the biciptal tuberosity, which was decompressed. The distal biceps tendon was nearly completely torn and repaired utilizing an Endobutton (Acufex, Smith and Nephew, Andover, MA) technique. A significant portion of the supinator muscle was noted to be torn off the proximal radius. No attempt at repair in this area was made as the injury involved friable muscle as opposed to tendon and conservative treatment was felt to be the most viable option. Concern, too, was for potential injury to the posterior interosseous nerve (PIN) with repair of the supinator.

After surgery, the patient was placed in a well-padded posterior long arm splint for 10 days, with the elbow at 90 degrees of flexion and the forearm in neutral position. At postoperative day 10, the patient was placed in a Bledsoe brace, and the ROM gradually increased. At four weeks, active ROM exercises were begun under the guidance of a physical therapist and at ten weeks, a program of progressive resistive exercise program initiated for flexion and supination/pronation at the elbow. At last followup, 18 months following surgery patient subjectively noted complete return of elbow strength without pain or deformity. Examination demonstrated full range of motion and normal resistive motor strength to both flexion and supination.

## 4. Discussion

Distal biceps rupture usually occurs after an unintentional or uncontrolled eccentric load to a flexed, shortened, and contracted muscle. The causes of the rupture are most likely multifactorial. A number of hypotheses have arisen to describe the pathogenesis: Degenerative, mechanical impingement, and ischemic factors appear to impact on the composition and structure of the tendons [8, 10]. Safran and Graham retrospective study concluded that smokers have a 7.5 times greater risk of rupture biceps when compared to nonsmokers [2].

In contrast to their male counterparts, there is limited information available regarding women with distal biceps tendon ruptures. Jockel et al. [7] described a series of 13 women with partial distal biceps tendon ruptures who underwent surgical repair; they concluded that women, unlike men, typically present with a partial tendon injury, contributing to the limited symptomatology and challenging initial diagnosis.

**Figure 3:**
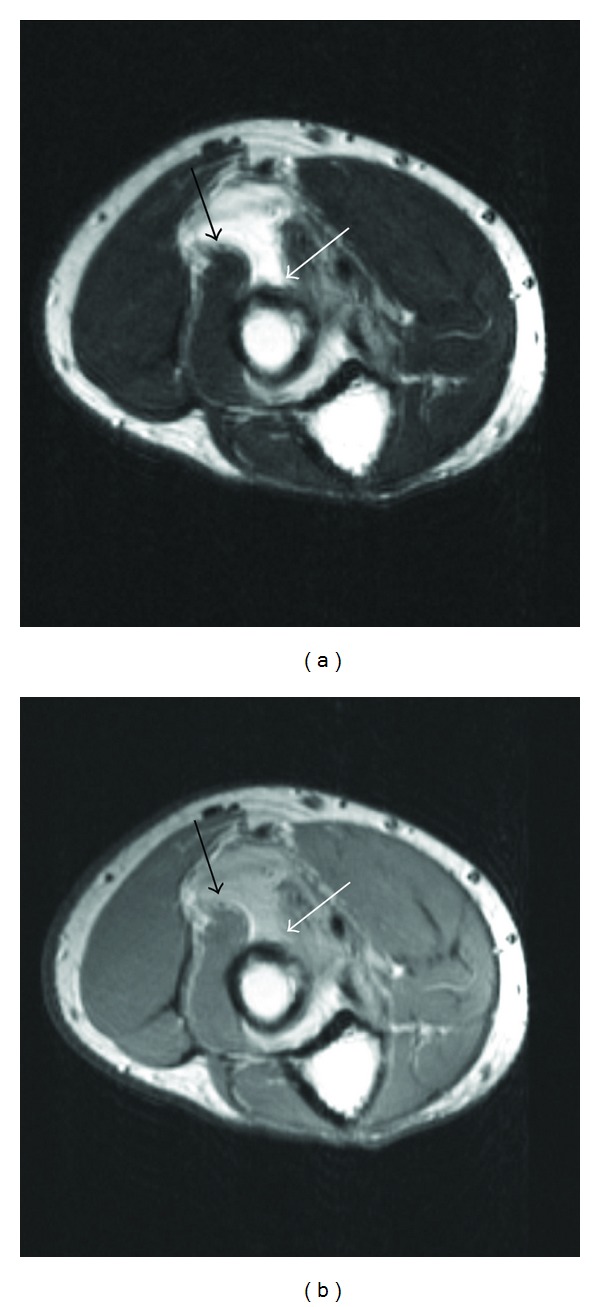
Axial T2 (a) and intermediate weighted (b) axial images at a slightly more distal level demonstrating partial tearing of the supinator muscle which appears to be peeled back off its medial attachment (solid white arrow) with the torn-free edge displaced anterolaterally (solid black arrow).

Diagnosing partial ruptures of the distal biceps can be challenging due to variable clinical presentations and subtle symptoms. Both MRI and ultrasound are useful tools for the diagnosis. The MRI images demonstrate thickening, increased signal, and irregular contour of the distal biceps tendon with edema or fluid in the peritendinous soft tissues and bicipitoradial bursa and occasional marrow edema or cystic changes at the radial tuberosity [11, 12] (Figures 3(a) and 3(b)). 

The supinator is a broad, flat muscle that spirals around the upper third of the radius. It originates from the lateral supracondylar ridge of humerus and posterior part of ulna and attaches to the outer surface of the upper third of the radius between the anterior oblique line and the posterior oblique ridge [11, 13, 14]. 

There are two major supinators on the forearm. One is the supinator muscle, which acts in unresisted supination of the forearm when the elbow is extended and holds the forearm in supination. The other is the biceps muscle, which acts in forceful supination on minimal elbow flexion. The supinator muscle also assists flexion of the forearm at the elbow when the forearm is held intermediate between supination and pronation through the fibers attached to the anterior capsule of the humeroulnar joint and epicondyle fibers [13, 14].

**Figure 4:**
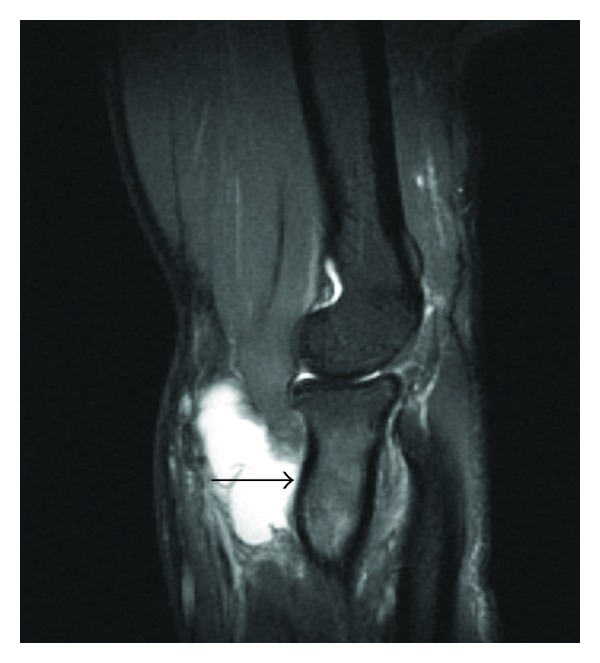
Sagittal STIR image of the elbow demonstrating a large exposed area of the anterior radial cortex where the supinator muscle has been stripped away (solid black arrow).

Isolated tears of the supinator muscle are rare. The diagnosis can be reached via clinical findings and ultrasound or MRI (Figure 4). Clinical differential diagnosis includes distal biceps tendon rupture, which could be physically examined using a biceps squeeze test (compression of the biceps brachii muscle belly, which generates tension in the distal biceps tendon and elicits supination of the forearm if the biceps tendon is intact) [15] and joint play and a speed test (where the patient's arm is placed in 90-degree forward flexion with the elbow extended and the forearm supinated, and the forearm is then pressed down against patient's active resistance). The sign is positive if pain is triggered in the anterior deltoid region [3]. A Heuter test (forceful flexion with the forearm pronated and biceps contracted simultaneously, causing supination of the forearm) is positive if forearm supination is not observed [3]. Positive results of the tests mentioned above favor distal biceps tendon rupture. Isolated supinator muscle tear is considered only when other possibilities such as distal biceps tendon rupture have been ruled out. In order to isolate the supinator muscle, the patient should be able to supinate the forearm while elbow is extended, therefore excluding the biceps, which is the major muscle involved in supination when the elbow is slightly flexed.

The authors postulate that this combined injury is extremely rare due to the need for combined eccentric contraction of both these muscle/tendon groups at the index moment of injury. The flexion angle of the elbow combined with the supination moment must be at a very precise position to cause the two tendons to avulse.

Baker and Bierwagen theorize that flexion and supination strength deficits cause a significant hindrance in activities of daily living, and that nonoperative treatment provides inferior outcomes when compared to surgical correction [5]. Conversely, Seiler et al. concluded that biceps tendon partial ruptures provided only limited functional loss with nonoperative treatment. They believe that the age and lifestyle of a patient are very important in determining the recommended treatment [10].

There are different surgical techniques and fixation mechanisms for repair of a distal biceps tendon rupture. Historically, single incision technique is associated with higher incidences of nerve injury [16, 17], and dual incision carries a higher risk of heterotopic ossification [18]. The advent of new noninvasive techniques for both approaches has significantly decreased their complication rate.

## 5. Conclusion

Distal biceps tendon rupture is a relatively uncommon occurrence in the general female population and, to our knowledge, has not been reported in association with a supinator muscle tear. We report a case of 51-year-old woman who experienced sharp pain in her forearm and elbow after lifting a heavy object. History and physical examination raised suspicion for a distal biceps tendon rupture. MRI imaging determined a combined distal biceps tendon tear with a supinator muscle tear with subsequent confirmation at surgery. Surgical repair was performed for the distal biceps tendon only through a single incision approach using the Endobutton technique. Different studies have shown that a single incision and dual incision techniques provide similar results, with each carrying its own risks.

##  Conflict of Interests

The authors declare that there is no conflict of interests.
